# Genomic correlates of programmed cell death ligand 1 (PD-L1) expression in Chinese lung adenocarcinoma patients

**DOI:** 10.1186/s12935-022-02488-z

**Published:** 2022-03-27

**Authors:** Kang Li, Jun Liu, Lin Wu, Yajie Xiao, Jia Li, Haijian Du, Zhikun Zhao, Chao Sun, Yongtian Zhao, Jie Yang, Dongfang Wu, Zhuxiang Zhao, Bolin Chen

**Affiliations:** 1 Thoracic Medicine Department 2, Hunan Cancer Hospital/the Affiliated Cancer Hospital of Xiangya School of Medicine, Central South University, Changsha, China; 2 Department of Respiratory Medicine, The First Affiliated Hospital of Jinan University, Guangzhou, China; 3 Department of Pulmonary and Critical Care Medicine, Guangzhou First People’s Hospital, School of Medicine, South China University of Technology, Guangzhou, China; 4 YuceBio Technology Co., Ltd., Shenzhen, China; 5 Department of Oncology II, Clifford Hospital, Guangzhou, China

**Keywords:** PD-L1 expression, Lung adenocarcinoma, Genomic profile, Immune signature, Immunotherapy

## Abstract

**Background:**

Although PD-L1 expression is a crucial predictive biomarker for immunotherapy, it can be influenced by many factors.

**Methods:**

A total of 248 Chinese patients with lung adenocarcinoma was retrospectively identified. Data for clinical features, gene alternations, signaling pathways and immune signatures was analyzed among negative expression group (TPS < 1%, n = 124), intermediate expression group (1% ≤ TPS < 50%, n = 93), and high expression group (TPS ≥ 50%, n = 38). Clinical outcomes among different expression groups were also evaluated from public database.

**Results:**

Firstly, high tumor mutation burden was significantly associated with high PD-L1 expression in these Chinese patients with lung adenocarcinoma. In addition, gene alternations including TP53, PRKDC, KMT2D, TET1 and SETD2 apparently occurred in high PD-L1 expression group. Moreover, pathway analysis showed that mutations involving in DDR pathway, TP53 pathway, cell-cycle pathway and NOTCH pathway were obviously varied among three PD-L1 expression groups. Besides, most of patients in high PD-L1 expression group from TCGA database were determined as high-grade immune subtypes (C2-C4), showing significant higher proportions of IFN-gamma, CD8+ T-cells, NK cells, NK CD56 dim cells, Th1 cells, Th2 cells (P < 0.0001). Moreover, *SETD2* mutation slightly correlated with overall survival from MSKCC cohort (HR 1.92 [95%CI 0.90–4.10], *P* = 0.085), and the percentage of IFN-gamma was significantly higher in *SETD2* mutant group than in wild-type group (*P* < 0.01).

**Conclusions:**

This study illustrated in-depth genomic correlates of PD-L1 expression in Chinese lung adenocarcinoma patients and relevant immune signatures from public database, which might interpret more potential molecular mechanisms for immunotherapy in NSCLC.

## Background

Non-small cell lung cancer (NSCLC) comprises about 80% of lung cancers, which are the major cause of cancer-related death [1]. Platinum-based chemotherapy or targeted therapy for specific driver genes used to be standard therapies for NSCLC, but drug related resistance to these treatments becomes huge challenges [2–5]. Immune checkpoint blockades (ICBs), including programmed death 1 (PD-1) inhibitors and cytotoxic T lymphocyte antigen-4 (CTLA-4) antibodies, have recently revolutionized the treatments for NSCLC and have emerged as promising therapeutic strategies for NSCLC patients [6, 7].

As there were still a certain number of patients who cannot benefit from ICBs, predictive biomarkers for clinical responses to the immunotherapies have provided clinical assistances for clinicians in early selection of those responders and timely implementation of therapeutic regimens [6, 7]. For example, some studies have demonstrated that positive programmed death-ligand 1 (PD-L1) expression level significantly correlated with an improved response in NSCLC [8, 9]. Based on the results of KEYNOTE-158 clinical trial, pembrolizumab has been approved by FDA as the front-line therapy for advanced lung cancer patients who present high PD-L1 expressions (TPS > 50%) and who are diagnosed as EGFR or ALK wild-type [10]. In KEYNOTE-042 clinical trial, front-line pembrolizumab therapy for metastatic NSCLC patients who have positive PD-L1 expression (TPS ≥ 1%) presents better clinical outcomes compared with platinum-based chemotherapy [11].

However, other studies showed low PD-L1 expression level in NSCLC (< 10%) cannot predict treatment response [8, 9]. PD-L1 expression has been found to be influenced by some factors like detection methodology and tumor heterogeneity in NSCLC [12]. In addition, alternations involved with TP53, KRAS, EGFR, ALK, STK11 and PTEN can affect PD-L1 expression [13–16]. Besides, activating of oncogenic signaling pathways including PI3K-AKT-mTOR pathway, JAK-STAT pathway and KRAS-ERK pathway, can also induce PD-L1 expression in NSCLC or in other cancer types [17–19]. As a result, prognostic value of this biomarker for ICBs was recently challenged. Alternatively, other biomarkers such as tumor mutation burden (TMB) and tumor infiltrating lymphocytes (TILs), have been validated for predicting the efficacy of ICBs [20, 21].

With advances of next-generation sequencing (NGS) techniques, we retrospectively conducted a in-depth analysis to characterize the factors associated with PD-L1 expression in Chinese lung adenocarcinoma patients. This study might help with illustrating potential molecular mechanisms of immunotherapy in NSCLC.

## Methods

### Study design

Patients with lung adenocarcinoma who were received anti-cancer treatments in our hospital from January 2019 to May 2020 was retrospectively identified and relevant clinical data were collected. Formalin-fixed paraffin-embedded (FFPE) tumor tissue or fresh tissue for each patient were either taken from a biopsy or surgery for PD-L1 expression assay and genomic profiling using NGS panel (YuceOneTM ^Plus^, Yucebio, China).

### PD-L1 immunohistochemistry

The Dako PD-L1 IHC 22C3 pharmDx assay was used to detect PD-L1 protein expression in FFPE slides according to the manufacturer’s recommendations. PD-L1 expression was calculated using tumor proportion score (TPS) according to the percentage of tumor cells with complete or partial membrane staining (central or marginal tumor region). Then, patients were divided as “negative” expression group (TPS < 1%), “intermediate” expression group (1% ≤ TPS < 50%), and “high” expression group (TPS ≥ 50%).

### Next generation sequencing and mutation analysis

Genomic profiling was performed on tumor tissue and matched peripheral blood samples. Genomic DNAs were isolated from tumor specimens and blood, and extracted using the GeneRead DNA FFPE Kit (Qiagen) and Qiagen DNA blood mini kit (Qiagen). Then, extracted DNAs were amplified, purified, and analyzed using NGS panel (YuceOne™ Plus, Yucebio, China).

Sequencing reads with > 10% N rate and/or > 10% bases with quality score < 20 were filtered using SOAPnuke (Version 1.5.6). The somatic single nucleotide variants (SNVs) and insertions and deletions (InDels) were detected using VarScan (Version 2.4), and further in-house method was applied to filter the possible false positive mutations. Then, SnpEff (Version 4.3) was used to perform functional annotation on the mutations detected in the tumor sample. Tumor mutation burden (TMB) was calculated using non-silent somatic mutations, including coding base substitution and indels.

HLA typing of tumor and matched control samples were assessed by OptiType (Version 1.3.2). The loss of heterogeneity (LOH) of HLA were detected by LOH HLA [22]. The neoantigen prediction was performed as previously described [23]. Tumor neoantigen burden (TNB) was measured as the number of mutations which could generate neoantigens per megabase.

### Copy number variations analysis

Somatic copy number alterations (SCNAs) analysis was performed using Allele-Specific Copy number Analysis of Tumors (ASCAT) with default parameters and FACETS algorithm. Then GISTIC2.0 was used to identify significant driver somatic CNVs by evaluating the frequencies and amplitudes of observed events. Chromosomal instability (CIN) was estimated using the weighted chromosomal instability (wCIN) score, which defined as the average of this percentage value over the 22 autosomal chromosomes [24].

### Pathways and immune signatures analysis

Genes in pathways analysis were compared with previously reported gene list [25, 26] and overlapping genes covered in the YuceOne™ Plus panel. Additionally, proportions of IFN-gamma signature and infiltrating immune cells were analyzed according to previous studies [27, 28]. The immune signature scores were calculated using ssGSEA method implemented by R package GSVA [29].

### Statistical analysis

Correlations between PD-L1 expression and clinical parameters were analyzed using the Fisher's exact test for categorical variables. Kruskal–Wallis rank sum tests were used for comparisons of continuous variables across multiple groups. Wilcox rank sum tests were used for comparisons of continuous variables between two groups. Multiple comparison corrections were used to calculate Q values by the FDR correction. Survival analysis was performed using Kaplan–Meier survival plot and log-rank test p value was calculated. P < 0.05 or Q < 0.25 were considered statistically significant. All statistical analyses were performed in the R Statistical Computing environment v3.6.1 (http://www.r-project.org).

## Results

### General clinical and mutational characteristics in Chinese lung adenocarcinoma patients

As shown in Table 1, a total of 248 Chinese lung adenocarcinoma patients were identified and included in this study. According to the results of PD-L1 expression essay, these patients were divided into three group, negative PD-L1 expression group with a TPS < 1% (n = 124, 50%), intermediate PD-L1 expression group with a TPS 1%-49% (n = 93, 38%), and high PD-L1 expression group with a TPS ≥ 50% (n = 38, 12%) (Table 1).

**Table 1:** Clinical characteristics of patients by PD-L1 expression groups

	Negative group (TPS < 1%)	Intermediate group (TPS 1%-49%)	High group (TPS ≥ 50%)	*P* value
N = 248	124 (50%)	93 (38%)	31 (12%)	
*Gende*r				
Male (n = 134)	65 (52%)	49 (53%)	19 (61%)	0.66
Female (n = 117)	59 (48%)	44 (47%)	12 (39%)	
*Age (years*)				
Median (range)	58 (23 ~ 84)	58 (27 ~ 80)	60 (32 ~ 73)	0.86
*TMB (Mut/Mb*)				
Median (IQR)	4.0 (2.01 ~ 5.36)	3.4 (2.68 ~ 6.70)	6.0 (4.02 ~ 14.41)	< 0.001*
*TNB (Neo/Mb*)				
Median (IQR)	1.3 (0.67 ~ 2.68)	1.3 (0.67 ~ 3.35)	2.7 (1.34 ~ 4.02)	0.09
*HLA LOH*				
Negative	98 (79%)	77 (83%)	21 (68%)	0.16
Positive	26 (21%)	16 (17%)	10 (32%)	

*P values < 0.05 are indicated in bold and italics

*TMB*: tumor mutation burden (*Mut/Mb* mutations per megabase); *TNB:* tumor neoantigen burden (*Neo/Mb* neoantigens per megabase); *HLA* : human leukocyte antigen; *LOH:* loss of heterogeneity; *IQR:* interquartile range

The median age and gender proportion was very similar among the three PD-L1 expression group, implying that PD-L1 expression level was not affected by either age or gender. The median TMB in PD-L1 high expression group was significantly higher than those values in intermediate or negative expression group [median (interquartile range) 6.0 mut/Mb (4.02–14.41) versus 3.4 mut/Mb (2.68–6.70) versus 4.0 mut/Mb (2.01–5.36), respectively; *P* < 0.001] (Table 1). Similarly, the median TNB in PD-L1 high expression group was higher than intermediate or negative expression group [median (interquartile range) 2.7 neo/Mb (1.34–4.02) versus 1.3 neo /Mb (0.67–3.35) versus 1.3 neo /Mb (0.67–2.68), respectively; *P* < 0.1]. However, HLA LOH was not associated with PD-L1 expression among the three PD-L1 groups (*P* > 0.1).

### Significant genomic mutations associated with PD-L1 expression in Chinese lung adenocarcinoma patients

**Fig. 1:**
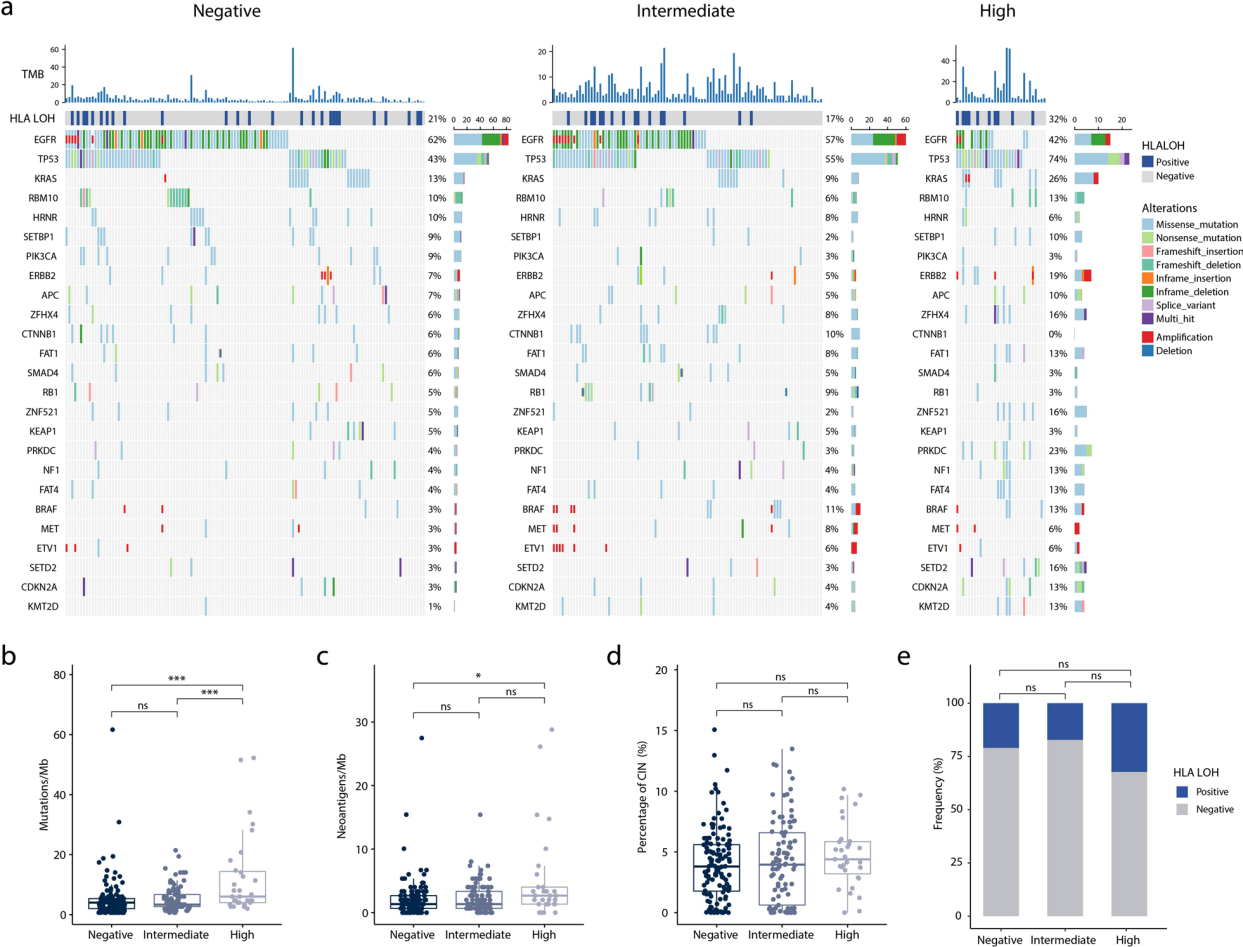
Distinct mutational patterns and biomarkers of Chinese lung adenocarcinoma patients grouped by PD-L1 expression. **a**) The mutational landscape of top 25 mutated genes. PD-L1 expression levels, TMB and HLA LOH status are annotated in the top panel. Associations between PD-L1 expression and immunotherapy related biomarkers including **b**) TMB, **c**) TNB, **d**) CIN and **e**) HLA LOH. TMB, tumor mutation burden. HLA, human leukocyte antigen. LOH, loss of heterogeneity. TNB, tumor neoantigen burden. CIN, chromosome instability. 'ns', not significant. *P < 0.05, ***P < 0.001

The 25 most frequently genomic alternations, such as high oncogenic amplifications or mutations and deep deletions in tumor suppressors, were listed in Fig. 1. These genomic alternations with a frequency more than 15 % included *EGFR* and *TP53* in PD-L1 negative expression group and PD-L1 intermediate expression group, *EGFR*, *TP53*, *KRAS*, *ERBB 2*, *ZFHX 4*, *ZNF 521*, *PRKDC* and *SETD 2* in PD-L1 high expression group. Among the three PD-L1 expression groups, there were significant enrichment diversities of gene mutation in *TP53* (74.2% versus 54.8% versus 42.7%, *P* < 0.01) and *KMT2D* (12.9% versus 4.3% versus 0.8%, *P* < 0.01). In contrast to intermediate or negative expression group, patients in PD-L1 high expression group were obviously enriched with genomic mutations in *PRKDC* (22.6% versus 3.2% versus 4%, *P* < 0.01), *SETD2* (16.1% versus 3.2% versus 3.2%, *P* < 0.05) and *TET1* (12.9% versus 4.3% versus 0.8%, *P* < 0.05). Besides, *BRAF* mutations significantly occurred in PD-L1 intermediate expression group than negative group (10.8% versus 3.2%, *P* < 0.05), while *KRAS* mutations apparently happened in PD-L1 high group than PD-L1 intermediate group (25.8% versus 8.6%, *P* < 0.05). Besides, EGFR mutations showed slight nagetive asocciations with PD-L1 expression in PD-L1 high group than intermediate or negative expression group (41.9% versus 57.0% versus 62.1%, *P* > 0.05).

### Key signaling pathways related with PD-L1 expression in Chinese lung adenocarcinoma patients

**Fig. 2:**
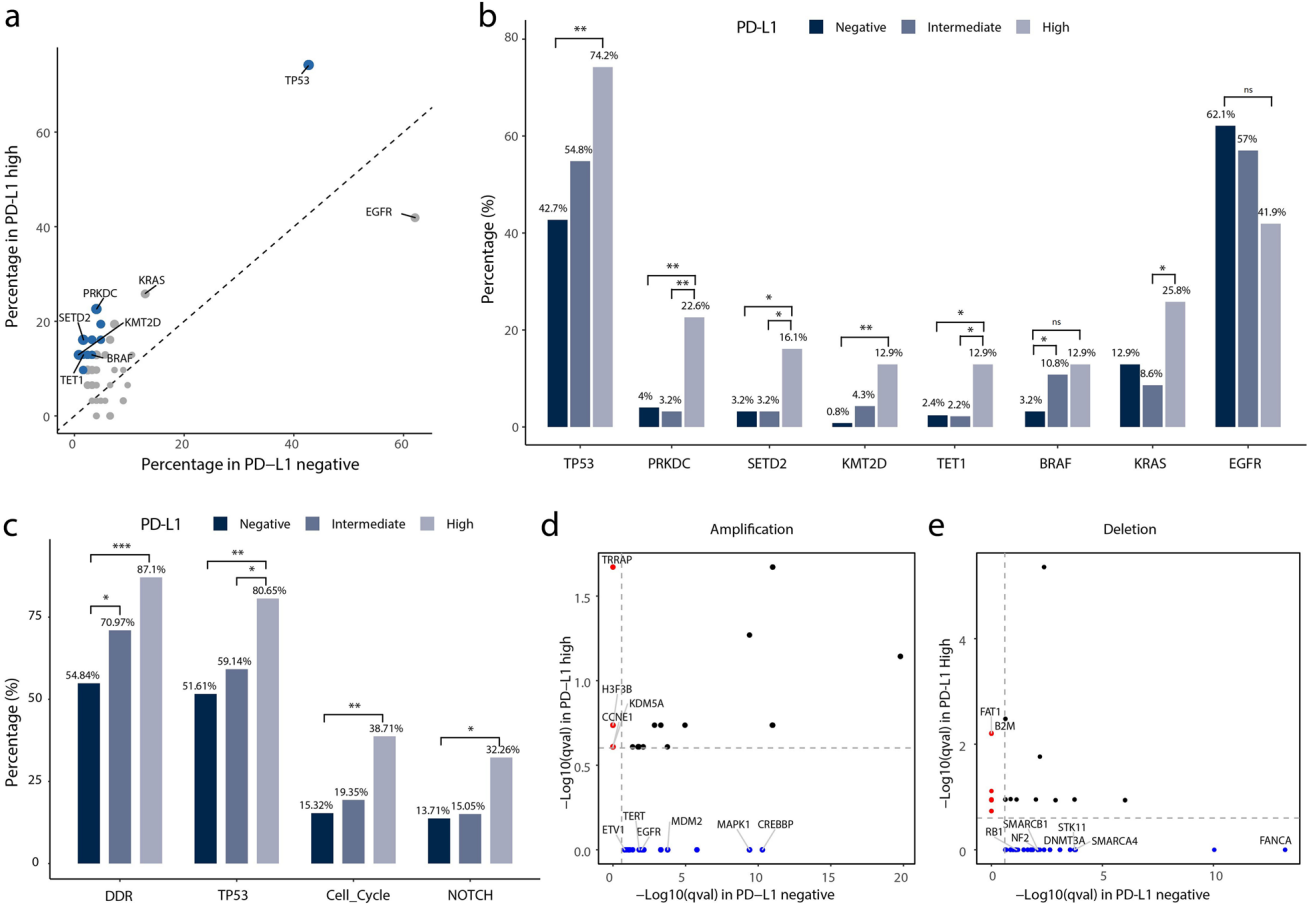
Associations between gene mutations and PD-L1 expression in Chinese lung adenocarcinoma patients. **a**) Percentages of mutated genes between PD-L1 high and negative expression groups. Blue dots denote genes associated with significantly differential PD-L1 expression (Q value < 0.25). **b**) Percentages of mutated genes among different PD-L1 expression groups. **c**) Percentages of significantly mutated pathways among different PD-L1 expression groups. **d**) Amplifications and **e**) deletions between PD-L1 high and negative expression groups. CNVs with Q value < 0.25 were significantly. Red dots denote CNV events only in PD-L1 high group. Blue dots denote CNV events only in PD-L1 negative group. CNVs, copy number variations. *P < 0.05, **P < 0.01, ***P < 0.001

**Fig. 3:**
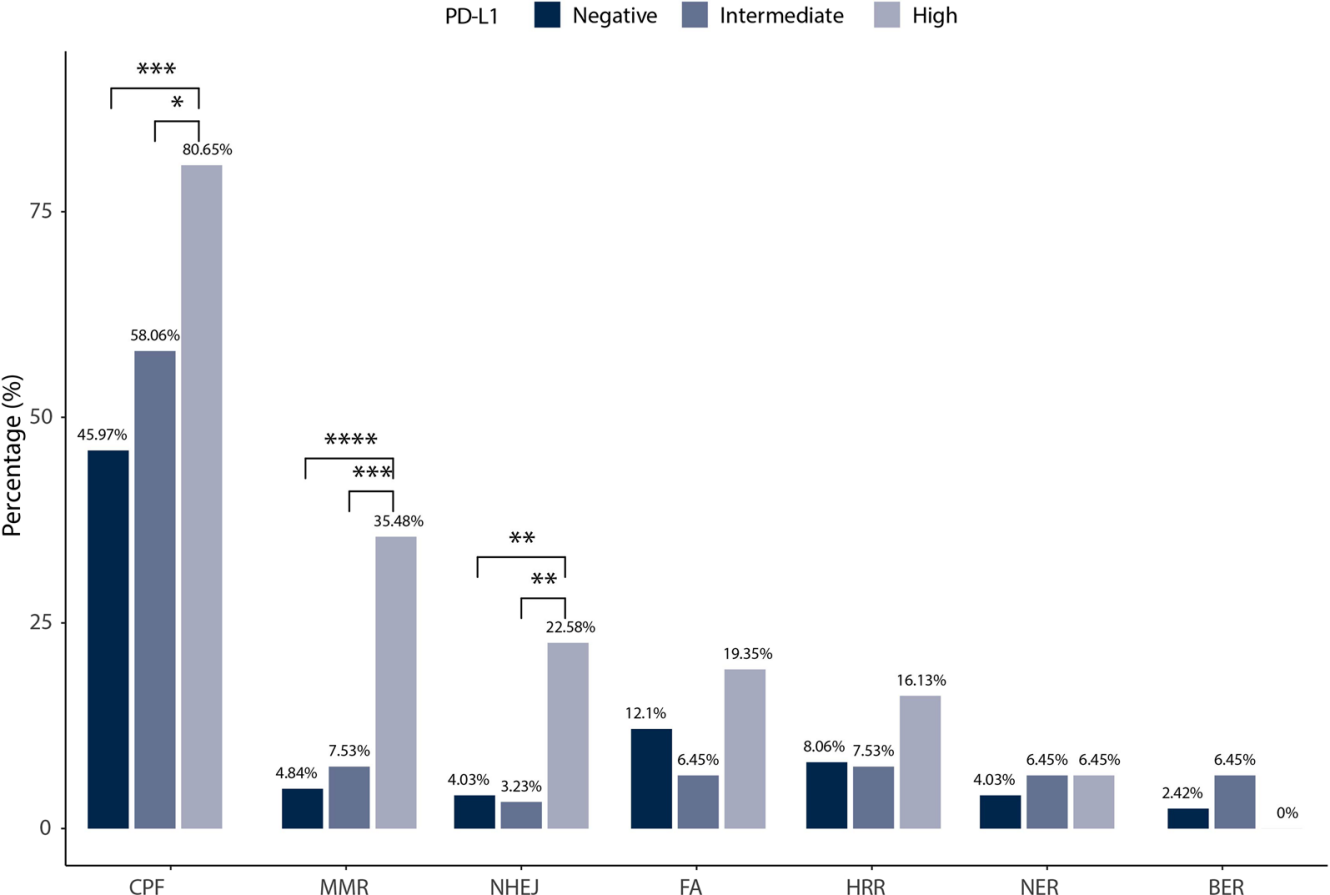
Percentages of mutated genes in DDR pathways among different PD-L1 expression groups. DDR, DNA damage response; CPF, check point factors; MMR, mismatch repair; NHEJ, nonhomologous end-joining; FA, Fanconi anemia; HRR, homologous recombination repair; NER, nucleotide excision repair; BER, base excision repair. *P < 0.05, **P < 0.01, ***P < 0.001, ****P < 0.0001

Next, we also performed further analyses in oncogenomic pathways in this study (Fig. 2). Alterations involved with the DNA damage response (DDR) signaling pathway happened less in PD-L1 negative expression group than PD-L1 high or intermediate expression group (54.84% versus 87.1% versus 70.94%, *P* < 0.05), due to the significant gene mutations in check point factor (CPF), mismatch repair (MMR), and nonhomologous end-joining (NHEJ) (Fig. 3). In PD-L1 high expression group, mutations in TP53 pathway were more frequently than PD-L1 negative expression group (80.65% versus 51.61%, *P* < 0.01) or intermediate expression group (80.65% versus 59.14%, *P* < 0.05). Besides, PD-L1 high expression group was more likely to have more gene mutations in cell cycle signaling pathway (38.71% versus 15.32%, *P* < 0.01) and NOTCH signaling pathway (32.26% versus 13.71%, *P* < 0.05) than PD-L1 negative expression group. Moreover, amplification of *TRRAP*, *H3F3B*, *KMD5A* and *CCNE1* and deletions of *FAT1* and *B2M* were distinctly enriched in PD-L1 high expression group than negative expression group. On the contrast, amplifications of *CREBBP*, *MAPK1 *, *MDM2*, *EGFR*, *TERT* and *ETV1* and deletions of *FANCA*, *SMARCA4*, *STK11*, DNMT3A, *SMARCB1*, *NF2* and *RB1* were obviously enriched in PD-L1 negative expression group.

### Major immune signatures linked to PD-L1 expression in lung adenocarcinoma patients from TCGA database

**Fig. 4:**
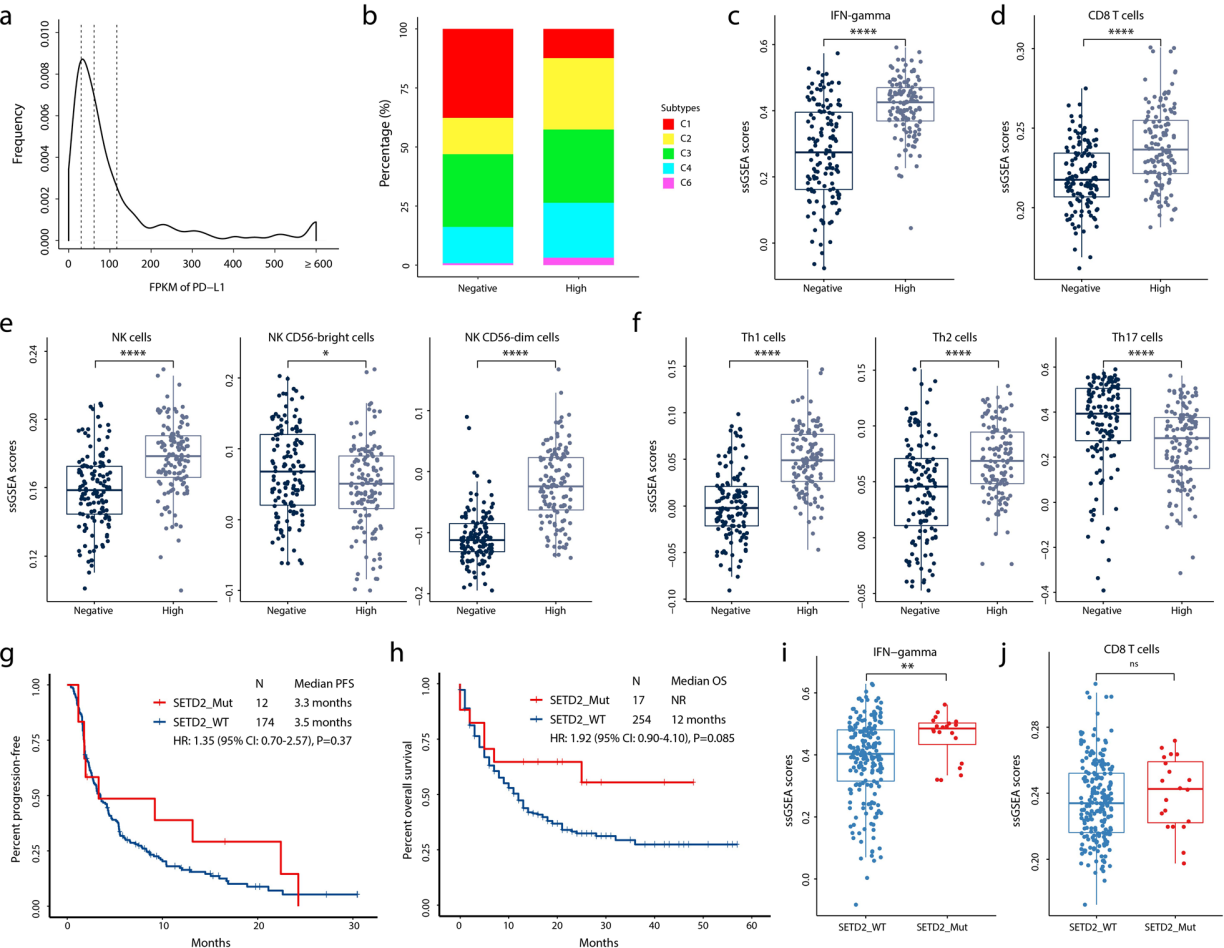
Characterization of immune signatures in public population. **a**) Distribution of PD-L1 expression in TCGA LUAD patients. The dashed lines denote 1st quantile, median and 3rd quantile. **b**) The percentages of immune subtypes in PD-L1 high and negative expression subgroups. The normalized ssGSEA scores of **c**) IFN-gamma, **d**) CD8+ T cells, **e**) NK cells and **f**) Th cells in PD-L1 high and negative expression subgroups. Kaplan–Meier estimates of **g**) progression-free survival (Rizvi cohort) and **h**) overall survival (MSKCC cohort) in the groups with or without SETD2 mutations. The normalized ssGSEA scores of i) IFN-gamma and j) CD8+ T cells in the groups with or without SETD2 mutations. LUAD, lung adenocarcinoma; IFN, interferon;NK cells, natural killer cells; Th cells, T helper cells; PFS, progression-free survival; OS, overall survival. ‘ns’, not significant. *P < 0.05, **P < 0.01, ****P < 0.0001

Based on TCGA-LUAD database, we primarily characterized immune signatures among different PD-L1 expression groups in Fig. 4 a–f and found that most of patients in high PD-L1 group were determined as high-grade immune subtypes (C2-C4). Compared with PD-L1 negative expression group, higher proportions of IFN-gamma, CD8+ T cells, NK cells, NK CD56 dim cells, Th1 cells, Th2 cells (*P* < 0.0001) and lower percentage of NK CD56 bright cells and Th17 cells (*P* < 0.05) was observed in PD-L1 high expression group, supporting that high PD-L1 expression level can be a prognostic marker for anti-cancer immunotherapy.

### Potential therapeutic response correlated to *SETD2* mutation from public cohort

As shown in Fig. 4g–j, the prognostic value of *SETD2* mutation was slight positive with overall survival from MSKCC cohort (HR 1.92 [95%CI 0.90–4.10], *P* = 0.085), but not progression-free survival among the patients from Rizvi cohort (HR 1.35 [95%CI 0.70–2.57], *P* = 0.37). Furthermore, the percentage of IFN-gamma was significantly higher in *SETD2* mutant group than in wild-type subgroup (*P* < 0.01).

## Discussion

Taking consideration of some published clinical trials, PD-L1 expression can help direct clinicians to choose single-agent immunotherapy for NSCLC patients with high PD-L1 expressions or combined chemo-immunotherapy for NSCLC patients with low PD-L1 expressions. But, due to constantly emerging of converse results, prognostic value of PD-L1 expression for ICBs was recently challenged [8, 9].

Except for the variabilities in immunohistochemical staining antibodies and heterogeneous expressions in different tumor site, PD-L1 expression has been found to be influenced by some extrinsic or intrinsic factors in NSCLC. In this study, we conducted a in-depth analysis in order to reveal latent gemoic or clinical correlates associated with PD-L1 expression in Chinese lung adenocarcinoma patients. In this retrospectively study, clinical features such as age and gender cannot affect PD-L1 expression in lung adenocarcinoma. High TMB levels were significantly as associated with high PD-L1 expression in lung adenocarcinoma (P < 0.05), which was consistent with those findings from multicenter studies [14, 15].

It is generally acceptable that patients from different ethnic groups have unique clinical features and oncogenic mutations in different cancers. Although some studies highlighted the molecular associations between genomic alternations of TP53, KRAS, EGFR and PD-L1 expression [13–16], similar studies focusing on Asian population are still very limited. Based on 15-gene NGS panel testing, Liu et al. found that EGFR mutations were more common in PD-L1 negative expression group (TPS < 1%), ALK mutations were more common in PD-L1 intermediate group (TPS 1%–49%), and BRAF and MET mutations were more common in PD-L1 high group ( TPS ≥ 50%) in Chinese lung cancer patients [30]. In addition to these common gene mutations, we revealed the obvious occurrences of genetic alternations in TP53, PRKDC, KMT2D, TET1 and SETD2 for high PD-L1 expression in Chinese lung adenocarcinoma patients. Similarly, it is recently reported that 75% of mutant PRKDC patients with lung cancers can response to immunotherapy, suggesting PRKDC can be explored as both a predictive biomarker and a therapeutic target for ICBs [31]. These results may enrich the mutational spectrum associated with PD-L1 expression in Chinese lung adenocarcinoma patients, and provide potential therapeutical target for immunotherapy in NSCLC.

Besides, activating signaling pathways like PI3K-AKT-mTOR pathway, JAK-STAT pathway and KRAS-ERK pathway, can regulate PD-L1 expression in many cancer types [17–19]. Recently, NSCLC patients with driver gene mutation in DDR pathways presented significant higher TMB values and higher objective response rate, longer median PFS after anti-cancer immunotherapy [32].We also found gene alternations for DDR pathway, TP53 pathway, cell cycles pathway and NOTCH pathway apparently happed in high PD-L1 expression patients (P < 0.05), which might provide more evidences for illustrating molecular mechanism involving with PD-L1 expression in NSCLC.

Due to the complexity of tumor immunity mechanisms, analyzing TILs in tumor microenvironments might be important for indicating tumor immunogenicity and predicting immunotherapy efficacy. Patients who were diagnosed as immune type I refer to those with high PD-L1 expression and CD8+ TLs in the tumor microenvironment, and most of these patients can benefit from ICIs [33, 34]. Also, these patients are likely to associate with increased numbers of somatic driver mutations or tumor neoantigen, and positive infection with Epstein-Barr virus, etc. [33, 34]. Likely, we primarily characterized immune signatures with PD-L1 expression in patients with lung adenocarcinoma from TCGA-LUAD database and found that the percentage of high-grade immune subtypes (C3-C5) in PD-L1 high group was higher than PD-L1 low group. Significant higher proportions of IFN-gamma, CD8+ T-cells, NK cells, NK CD56 dim cells, Th1 cells, Th2 cells were found in PD-L1 high group (*P* < 0.0001), whereas substantial lower percentage of NK CD56 bright cells and Th17 cells was observed (*P* < 0.05). Then, we found SETD2 mutation were slight positive correlated with overall survival from MSKCC cohort (HR 1.92 [95%CI 0.90–4.10], *P* = 0.085), and the percentage of IFN-gamma (*P* < 0.01) and CD8+ T-cells (*P* < 0.05) was higher in SETD2 mutant group than in wild-type subgroup.

This study involved several limitations. First, most of the patients in our studies were treatment-naïve for any anti-cancer therapy, which might present lower PD-L1 expression levels than after-line patients. Second, missing of some clinical diagnostic data like cancer stage and tumor site may lead to a less detailed analysis on the clinical impact on PD-L1 expression. Third, due to lack of clinical survival data like PFS and OS, we used TCGA data to evaluate the influence of PD-L1 expression on clinical response. Therefore, there were an inconsistence between stratifying patients by TPS in our study and by a quartile method in TCGA database. Besides, the current sample size might be small for patients with common drive genes like ALK and EGFR when investigating on the roles of these gene mutations on PD-L1 expression. These may cause some statistical bias finally. Further study with larger sample size are planned in the future.

## Conclusions

In summary, our study illustrated a clearer genomic landscape in Chinese lung adenocarcinoma patients of PD-L1 expression and relevant immune signatures from public database for interpreting the potential molecular mechanisms for clinical immunotherapy in NSCLC.

## Data Availability

The datasets used and analyzed during the current study are available upon reasonable request.

## Abbreviations

FFPE: Formalin-fixed paraffin-embedded
IN: Chromosomal instability
CNVs: Copy number variations
CPF: Check point factor
DDR: DNA damage response
LOH: Loss of heterogeneity
MMR: Mismatch repair
NHEJ: Nonhomologous end-joining
NGS: Next-generation sequencing
NSCLC: Non-small cell lung cancer
PD-L1: Programmed cell death ligand 1
SCNAs: Somatic copy number alterations
TILs: Tumor infiltrating lymphocytes
TMB: Tumor mutation burden
TNB: Tumor neoantigen burden
TPS: Tumor proportion score
wCIN: Weighted chromosomal instability
